# Understanding Dilated Mathematical Relationship between Image Features and the Convolutional Neural Network’s Learnt Parameters

**DOI:** 10.3390/e24010132

**Published:** 2022-01-16

**Authors:** Eyad Alsaghir, Xiyu Shi, Varuna De Silva, Ahmet Kondoz

**Affiliations:** Institute for Digital Technologies, Loughborough University London, Queen Elizabeth Olympic Park, Here East, London E20 3BS, UK; e.alsaghir@lboro.ac.uk (E.A.); v.d.de-silva@lboro.ac.uk (V.D.S.); a.kondoz@lboro.ac.uk (A.K.)

**Keywords:** CNN, causality, understandability, image features, excitation weights, ANOVA

## Abstract

Deep learning, in general, was built on input data transformation and presentation, model training with parameter tuning, and recognition of new observations using the trained model. However, this came with a high computation cost due to the extensive input database and the length of time required in training. Despite the model learning its parameters from the transformed input data, no direct research has been conducted to investigate the mathematical relationship between the transformed information (i.e., features, excitation) and the model’s learnt parameters (i.e., weights). This research aims to explore a mathematical relationship between the input excitations and the weights of a trained convolutional neural network. The objective is to investigate three aspects of this assumed feature-weight relationship: (1) the mathematical relationship between the training input images’ features and the model’s learnt parameters, (2) the mathematical relationship between the images’ features of a separate test dataset and a trained model’s learnt parameters, and (3) the mathematical relationship between the difference of training and testing images’ features and the model’s learnt parameters with a separate test dataset. The paper empirically demonstrated the existence of this mathematical relationship between the test image features and the model’s learnt weights by the ANOVA analysis.

## 1. Introduction

Recent advances in neural networks (NN) have enabled many strenuous tasks to be accomplished by machines, sometimes surpassing human performance. Object recognition [1], biometric recognition [2,3], scene understanding [4,5], image super-resolution [6], and image captioning [7] are a few of such machine intelligence tasks related to visual perception. In supervised visual perception tasks, machines can learn from repeated measurements of studied phenomena and the associated frequency of different event outcomes. Such visual perception tasks are at the heart of artificial intelligence (AI). Different recognition techniques, such as classification, retrieval of temporal features from time series data, and spatial information verification, require a measure of functional similarity between temporal and/or spatial information included within the input data [8].

However, the human’ brain categorises things according to what the brain is “looking for” when it sees the things [9]. In other words, the categorisation depends on what image features the brain sees during its analysis or focusing process [10]. This “looking for” mechanism enables humans to allocate a related recognition model effectively, although it needs, sometimes, further information (features) for effective analysis. This need can be noted when humans use other features, such as memory, smell, and location, in parallel with the initial object image for a faster recognition procedure. In 1943, Walter Pitts et al. created the first neural network computational model based on a mathematical algorithm known as threshold logic [11]. This algorithm was the primary key in developing the machine’s ability to generate recognition models. As a result of these models, a unique implementation was developed in convolutional neural networks (CNNs) during the last decade. The CNN analyses visual images to prove a decisive success in various computer vision tasks, such as video action recognition [10], image classification [12], image reconstruction [13], and segmentation [14]. However, there is a significant concern about the need for human interference in nearly all machine learning systems that deal with this technology field [15].

To address the gap in building a machine that can learn how to learn, we need to investigate any hidden mathematical link between the learnt parameters of the recognition system and the extracted features of a data point. Then the system can use this link at a pre-processing stage to further analyse a new object. With this development, it is possible to research new techniques for exploring the effects of image features on a model’s weight algorithmically. However, this new paradigm comes with severe challenges in adapting the current machine learning methods due to the many AI models, architectures, and feature-extraction techniques developed so far.

Despite much research on the causality [16] and understandability of AI [17,18], relatively little work has been performed to clarify the actual relationship between the extracted features of a trained object and the learnt parameters of a trained model. We will use “features” for the extracted features of an object and use “parameters” or “weights” for the learnt parameters of a model for simplicity of description in the paper hereafter. We hypothesise a relationship between the parameters and the features (i.e., excitation weights in a neural network [19]), and investigate this relationship by applying the ANOVA analysis [20]. This work is instrumental in increasing the recognition of objects in different classes by widening the neural network analysis abilities to align with the rapid development of big-data technology and high-performance computers.

## 2. Related Work

Humans identify unknown objects with their instinct to learn about these objects until they build a corresponding knowledge at the end, incorporating a lifetime of experience [21]. It was noticed that CNN’s filter dimensions are relatively fixed, aligning with a dramatic decrease in the number of the unique excitation weights along the CNN recognition technology development during the last decade [22]. This reflects a practical link between the number of the excitation weights and the model’s accuracy of performance with developed computations techniques and physical resources. Hence, deep learning becomes the best tool that yields good accuracy in image classification with large input compared to traditional computer vision algorithms [23]. These findings encouraged Poojary and Pai [24] to perform a comparative study of model optimisation techniques in fine-tuned CNN models, which concluded that fine-tuned transfer learning CNN models are best for building models with similar given tasks to the original task. However, they found that the CNN recognition accuracy is highly affected by the model optimisation techniques tested in their work.

The basic CNN computations are built on multi-dimensional dot products between the feature vectors and the model’s weight vectors [25]. Hegde et al. [26] studied how weight repetition during CNN inference could be used to improve the performance and saves energy according to the weights’ sparsity. This repetition formed the primary purpose for proposing the unique weight CNN accelerator (UCNN). By exploiting the repetition in all zero weights, the UCNN improved the efficiency of memory and energy, speeding up the computation processes to 3.7 times on three trendy CNNs. This approved the actual relationship between the model’s performance of accuracy and the input features.

Moreover, Han et al. [27] researched the intensity of neural network computation processes and memory usage to overcome the deployment difficulties on embedded systems. To reduce the overall computation costs, they studied the relationship between the model’s weights and the input training process. They addressed this issue by overcoming the fact that the CNN architecture is fixed during training, and proposed a three-step method to learn the essential connections by trimming the unimportant connections and retraining the network for weights fine-tuning. They decreased the number of weights by 9–13 times for various CNN networks without suffering or affecting the accuracy.

The work in this paper was inspired by the idea that a mathematical relationship exists between the resulting model’s parameters and the model’s featured input based on the related work. We proposed an empirical approval for this assumption to further develop in this area and to understand the model’s learning process better in the future.

## 3. Materials and Methods

We start our investigation by understanding the proposed assumption, the methodology of the empirical proof and experiments setups of the mathematical linkages, and the description of ANOVA analysis.

### 3.1. The Proposed Assumption

The CNN model’s learnable parameters are generally the weights and biases of any standard fully connected layer considering the model’s architecture and the calculation effects of different optimisation methods. Each layer’s parameters are generated, added up, and multiplied by input weights to obtain the entire network’s learnable parameters. These weights connect neural network variables similar to the coefficients of any standard regression model, which describe the relationship between the variables. They dictate how the related information influence is processed within the network such that the weights suppress the non-relevant input variables to a response variable. However, the significant difference between the regression and NN models is the number of excessive weights in the NN models. This characteristic helps add flexibility to neural networks for modelling non-linear functions through multiple interactions, with specific variables for interpreting challenging effects. Then, in developing the recognition models used in deep learning, the model is built upon bringing the prediction loss function to a minimum during the training and validation processes. This goal was achieved by applying a dot-product between each object’s features vector and the model’s weights vector, generating the model’s output (i.e., the predicted result).

The dot-product, denoted as (·), of two vectors $A=\left[a_{1},\;a_{2},\;\cdots ,\;a_{N}\right]$ and $B=\left[b_{1},\;b_{1},\;\cdots ,\;b_{N}\right]$ is the sum of the products of corresponding elements [28], as shown in (1):

(1) $$A\cdot B=\sum_{i=1}^{N}a_{i}\times b_{i}$$

Learning the weights relies on many factors such as the number of weights related to the CNN model’s architecture, the location of the weights within the model at the input of different layers, the initialised weight values that the training procedure started from, and a few other critical optimisation factors [29]. However, several algorithms were developed in recent deep learning techniques, trading computation accuracy for computational efficiency improvement. Some of these methods showed improvements in generalisation due to the noise effects of low-precision computations [30]. The practical implementation for an object prediction model is based upon applying the excitation operation according to the features to generate the final output with the minimal overall loss value [31]. This excitation operation is applied to generate the model’s weights for each feature channel with parameters, where these parameters learn to model the effective correlation between the feature channels. Therefore, the excitation operation must be flexible to learn the non-linear interactions and the non-mutually exclusive relationship between channels to capture the channel wise dependency [32]. Hence, during the development of deep learning technology, it was proved that the updates on a model’s excitation weights are mainly related to training the samples’ extracted feature values, assuming an overall mathematical relationship between them. Therefore, the selection of image features primarily affects the object’s retrieval performance [33].

This assumption raised the awareness of researching this mathematical relationship to find potential computational savings without a trade-off in accuracy. Therefore, we investigated this assumption and empirically proved it by: (1) building correlation values that reflect this relationship, and (2) applying the dot-product operation to different models’ parameters and different images’ features. Then we assessed the differences between the means of the generated values, and evaluated the variances of the groups’ means in different scenarios with the ANOVA analysis. The CNN models used in these scenarios were built with known or unknown and related or unrelated datasets to the model. The known datasets have been used in the model training or validation, and the unknown dataset has never been used in either training or validation. A related dataset is a dataset that belongs to the same domain as the training dataset, and an unrelated dataset does not belong to the domain of the training dataset of a model.

### 3.2. Methodology of Empirical Proof of the Assumption

This approach proves a mathematical relationship between the images’ features and the trained CNN model’s weights. We started with building correlation values between the features and the weights by applying the dot-product operation on them for each input image’s features.

Let’s denote, ${ℱ}_{i,k_{n}}^{r}$ as the *n*th element of feature vector ${ℱ}_{i,k}^{r}$ for image, i, of the Model k, within test dataset r, and denote $W_{k_{n}}$ as the *n*th element of the weight vector $W_{k}$ of Model k. The dot-product of the equal-sized vectors features, ${ℱ}_{i,k}^{r}$, and the weights, $W_{k}$ is shown in (2):

(2) $${ℱ}_{i,k}^{r}\cdot W_{k}=\sum_{n=1}^{N}({ℱ}_{i,k_{n}}^{r}\cdot W_{k_{n}}),$$

where *N* is the total number of elements in the vectors and equals 25,088.

We extract three different sets of weights, $W_{x,k_{n}},\;\mathrm{for}\;x\in \left[1,3\right]$, from the learnt weights of Model k. Then we obtain a single value $V_{i,k}^{r}$ of feature-weight (FW) component by calculating the average of the three dot-product values, $({ℱ}_{i,k}^{r}\cdot W_{k_{x}})$ for each image i in test set r for Model k, as shown in (3):

(3) $$V_{i,k}^{r}=\frac{\sum_{x=1}^{3}{ℱ}_{i,k}^{r}\cdot W_{x,k}}{x}$$

Then, we assess these component values for each tested dataset through seven different scenarios arranged in three categories to better understand the possible causality. These scenarios have been summarised in Table 1, which lists all experiments conducted.

**Figure 1:**
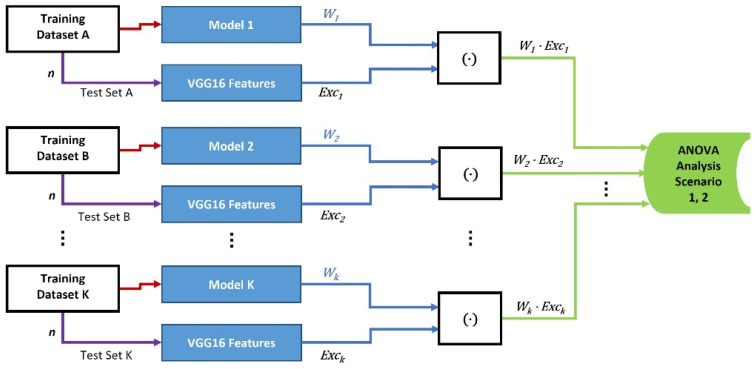
Diagram of Category 1 for the effect of training dataset sizes on the weights of models. In scenario 1, the small training dataset has 20 images, the number of test images n=20, and the number of models k=11. In scenario 2, n=400, k=3, and the size of training datasets are 10,000, 8000, and 1300, respectively. The generated FW vectors are [*n* × 1] in size.

In the first category, we assess whether the FW value is affected by the training dataset size using the ANOVA analysis. As shown in Figure 1, the FW values are built with 11 different models’ weights and their related (known to the model) input images’ features in the first scenario. The models are trained with a small size dataset of only 20 images. Then we apply the ANOVA one-way analysis on the FW values to examine whether they have similar mean values between the 11 different models. Three different models, Model 1 (M1), Model 2 (M2), and Model 3 (M3), are then trained with three large training datasets in the second scenario. This is used to test the null hypothesis of having similar mean values between the three different models’ FW values [20].

**Figure 2:**
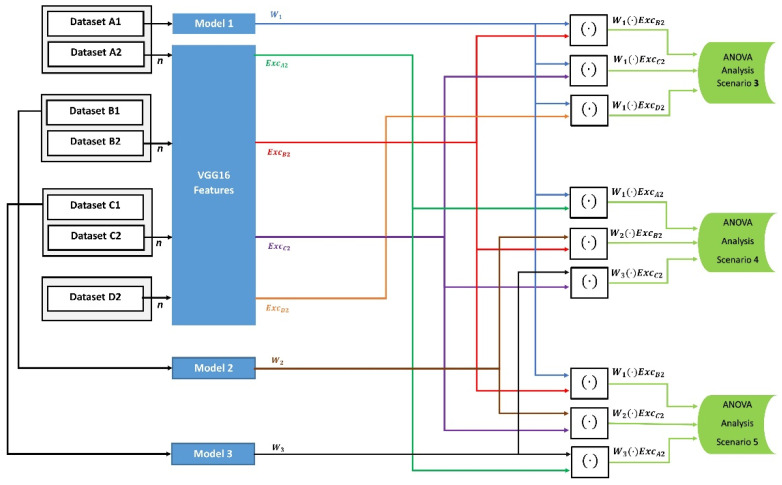
Diagram of Category 2 for the effect of unknown-unrelated and unknown-related datasets on the models’ weights. Models 1, 2, and 3 were trained with a large training dataset of 10,000, 8000, and 1300 images, respectively. The test dataset size n=200 in the third scenario with Model 1, and n=400 with the three models in the fourth and fifth scenarios. The generated FW vectors are [*n* × 1] in size.

In the second category, we assess if the FW variance is affected by the types of tested datasets. As shown in Figure 2, we train model M1 with a large training dataset that contains 10,000 images in the third scenario. In this scenario, the FW values for comparison are generated with three different test sets of images that are unrelated to the training classes and not used in training. Then we test whether the mean values between the FW components of the three tested unrelated datasets were similar or not. Moreover, we train three models, M1, M2, and M3, with three large datasets, and generate FW values with three related-unknown test datasets in the fourth scenario and three unrelated-unknown test datasets in the fifth scenario. Then, we apply the ANOVA one-way analysis in scenarios 4 and 5 to examine whether the FW values have similar mean values between the three models.

**Figure 3:**
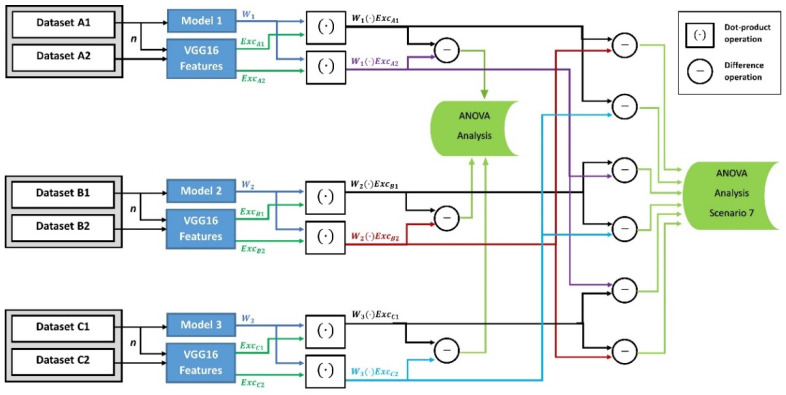
Diagram of Category 3 for the difference in FW values created between training and test datasets. In the sixth scenario, the FW difference between known-related and unknown-related test sets are tested. The difference between known-related and unknown-unrelated test sets are tested in the seventh scenario. The three models were trained with a large dataset of 10,000, 8000, and 1300 images, respectively, and tested with n=200 images per dataset. The generated FW vectors are [*n* × 1] in size.

Finally, in the third category, we assess if the variance of the FW differences is affected by the types of test datasets and training datasets. As illustrated in Figure 3, we developed a different deviation tendency measure for this category. In the sixth scenario, we generated the FW values between the three models’ weights and the features of both the known-related and unknown-related test datasets. Then we calculated the differences between these FW values for the ANOVA analysis. In the seventh scenario, and similar to the deviation tendency measure in the sixth scenario, we generated the FW values between the three models’ weights and features of both the known-related and unknown-unrelated test datasets. Then we calculated the differences between these FW values for the ANOVA analysis.

#### 3.2.1. Experiment Setup

The empirical proof has been implemented with Python 3.8.5 and TensorFlow 2.3.1 on a PC with an Nvidia Quadro M4000 GPU card. In all experiments, we used the same CNN model architecture with kernel size (1, 1), stochastic gradient descent (SGD) optimiser, categorical cross-entropy loss function, and rectified linear unit (ReLU) activation function. We also used the same CNN/VGG16 algorithm for feature initialisation and extraction. The experiments were applied with three different datasets. The first model, M1, was adapted to distinguish between cats and dogs from the Dogs vs. Cats dataset of 25,000+ images available on the Kaggle website [34]. We selected 20 images randomly for the small-sized dataset and 10,000 images for the large-sized dataset. However, the second model, M2, distinguishes between two types of facial emotions (happy and sad) using the AFEW2.0 dataset after cropping each image frame’s faces [34,35]. We selected 8000 images randomly for training and validation from this face dataset. The third model, M3, was trained with 1300 images to classify cars and flowers. The images were extracted from the Natural Images-Kaggle dataset of 6899 images from 8 distinct classes [36]. The Python code for the experimentation is published in GitHub [37].

As described in the following sections, we built the experiments through seven different setup scenarios in three categories.

- In the first category, we assessed the FW values of small size training datasets of 20 images applied to 11 identical CNN models in scenario 1, and large size datasets of 10,000, 8000, and 1300 images for training model M1, M2, and M3, respectively, in scenario 2. We built the FW components in the first scenario by applying the dot-product operation upon the features $Exc_{K}$ of eleven known-related test sets of 20 images and the weights $W_{k}$ of eleven models. In the second scenario, the FW components were built by applying the dot-product operation upon the features $Exc_{k}$ of 400 randomly selected known-related test images and the weights $W_{k}$ of three different models trained with the three large datasets. The two scenarios are illustrated in Figure 1.
- In the second category, we assessed the effect of different test set types on the FW values. We used a large training dataset of 10,000 images for training Model 1 in the third scenario. However, we used 10,000, 8000, and 1300 images for training Model 1, 2 and 3, respectively, in the fourth and fifth scenarios. In the third scenario, the dot-product operation was applied to model M1 with three unknown-unrelated test sets of 200 images. In the fourth scenario, the dot-product operations were applied with three unknown-related test sets of 400 images. Finally, the dot-product operations were applied in the fifth scenario with three unknown-unrelated test sets of 400 images. The three scenarios in Category 2 are shown in Figure 2.
- In the third category, we assessed the closeness between the FW values according to the mathematical difference tendency measure developed in this paper. In this measure, we used large training datasets to train three CNN models, with 10,000 for Model 1, 8000 for Model 2, and 1300 for Model 3. The difference measure was generated on two different scenarios for the ANOVA analysis. First, in the sixth scenario, the mathematical difference was calculated between the dot-product values that were generated on two cases: (1) between the features of the known-related test sets and the weights of the trained models, and (2) between the features of the unknown-related test sets and the weights of the trained models. Second, the mathematical difference was calculated between the dot-product values that were generated on another two levels: (1) between the features of known-related test sets and the weights of the trained models, and (2) between the features of unknown-unrelated test sets and the weights of the trained models, in the seventh scenario. The sixth and seventh test scenarios are shown in Figure 3.

#### 3.2.2. ANOVA Test Setup and the Null Hypothesis

With the ANOVA analysis, we assess the variance between two or more sets of values by splitting the observed aggregated variables in each test dataset into systematic and random factors. The systematic factors have a statistical effect on the tested dataset, while the other random factors do not. The null hypothesis (Null-H) is that all the observations have the same mean. This assessment can be performed by testing if the ANOVA probability value, *p*, is more than the cut-off acceptance value α, and the calculated F_Score is less than the ANOVA’s critical value F_Factor according to the given α [38]. Moreover, the α value is usually selected in the confidence intervals in the ANOVA test, as the lower the value of α, the wider the confidence interval. In our experiments, the minimum achieved *p* was 0.07 for failing to reject the null hypothesis in place. Therefore, we selected the value of α=0.05 due to its accepted result in the coherence of the tested results that we conducted.

In the first category, the null hypothesis is that the means between the FW values are similar. Failing to reject (i.e., accepting) the null hypothesis means a recognised resemblance between the FW values. This proves the existence of a mathematical relationship between the features of any known images and the weights of each related model, regardless of the model being trained with a large or small dataset.

However, with the second category, the ANOVA one-way test was built on the FW components between each model’s weights and the features of unknown test images. We explored the FW relationship linking one model’s weights and unknown-unrelated test sets in the third scenario. However, in the fourth and fifth scenarios, we explored the FW values between three trained models’ weights and unknown-related and unknown-unrelated test sets, respectively. This analysis enabled us to assess the null hypothesis of same FW means between the trained models and unknown test datasets. Rejecting the Null-H means no actual weights-features mathematical relationship, between any unknown test set and the model’s learnt weights.

With the third category, the ANOVA analyses whether the mean values of differences between all tested FWs are close to each other. In the sixth scenario, the differences are calculated between the FW values of known-related and unknown-related test datasets. In this case, accepting the null hypothesis proves a correlation in the mathematical difference between the FW values of known-related images and the unknown-related images. However, in the seventh scenario, the differences are calculated between two correlated FW values: (1) the FW values of large test sets presented to the models during the training and related to the domain of the trained model, and (2) the FW values of large test sets that have never been presented to the models during training or validation and unrelated to the domain of the trained model. In this case, the rejection of the null hypothesis proves no correlation in the differences between the FWs of the known-related images and the unknown-unrelated images.

## 4. ANOVA Test Results and Analysis

Here, we will describe the obtained results. We will start with the results obtained with the ANOVA analysis related to each test category. Then, we will go deeper into analysing the results to understand them better.

### 4.1. Results

**Figure 4:**
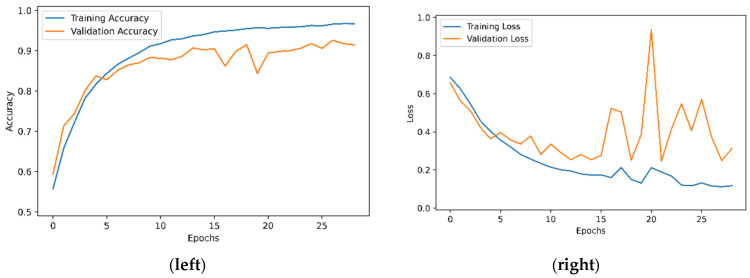
Performance of accuracy (**left**) and loss (**right**) for Model 1 in training and validation. Model 1 was trained with 1000 images from the Kaggle Dogs vs. Cats dataset.

All models are first trained according to the experiment conditions listed in Table 1. As an example, Figure 4 shows the performance of the Model 1 in accuracy and loss in training and validation for 30 epochs, with the model being trained with 10,000 images from the Kaggle Dogs vs. Cats dataset [34].

The ANOVA test results are shown in Table 2, where the calculated F_Score, the critical F_Factor, and the ANOVA test output are listed for all scenarios for the three test categories. In the first category, we have researched the effect of the training dataset size on the FWs. In the first scenario, the experiment results are organised according to the ANOVA results with all known-related 20 images features correlated with each tested model’s learnt weights. However, in the second scenario, we explored the FW components generated between 200 images (known-related) per class and the models’ parameters.

The ANOVA results of the effect of the test dataset types on the FWs between the test images’ features and the model’s weights are listed in the second category in Table 2. In Scenario 3, the ANOVA one-way analysis results are obtained with 200 unknown-unrelated images and each model’s weights. In Scenario 4, the results are generated between 400 known-unrelated images and the model’s weights. For Scenario 5, the results are generated between 400 unknown-unrelated images and the model’s weights. As it showed in the table, the null hypothesis is rejected in all scenarios in the second category by the ANOVA analysis.

We further researched the mathematical differences between different types of test sets to analyse the relationship between different FW components in the third category. The sixth scenario has generated the FWs between 200 images (unknown-related) and the model’s weights. As a result of this test in Scenario 6, the ANOVA analysis failed to reject the null hypothesis. Finally, in Scenario 7, we assessed the mathematical difference of the FW values when the dot-product operation was applied to the model’s learnt weights and the features of unknown-unrelated and known-related images. The ANOVA test rejected the null hypothesis for this scenario.

As shown in Table 2, regardless of whether the related images were known or unknown to the models in the test, the ANOVA analysis of Scenario 1, 2, and 6 failed to reject all null hypotheses, reflecting a mathematical relationship between the training dataset features and the model’s learnt weights. However, in the other scenarios, we achieved that the ANOVA’s analysis rejected the null hypothesis, reflecting no mathematical relationship between the learnt weights and any dataset unrelated to the trained model’s domain.

### 4.2. Analysis of Results

Throughout the assessed scenarios in the first category, all experiments generated overall *p* values larger than the cut-off acceptance value of α=0.05, and F_Score values smaller than the F_Factor value. Failure to reject the null hypothesis approves the FW relationship in the case of known-related test data sets due to no significant difference between the FW values. This finding proves that the trained model’s weights are mathematically related to and affected by the features of the known trained dataset, regardless of the size of the dataset.

Furthermore, out of the second category of tests where the unrelated test datasets are applied to the models, all ANOVA analysis return the results of *p* value being smaller than the cut-off acceptance value of α=0.05, and the F_Score values being larger than the F_Factor value. These results support rejecting the null hypothesis due to the significant difference between the assessed FW values. These rejections proved there was no mathematical relationship between the trained model’s weights and any unrelated test dataset’s features.

In Category 3, the ANOVA analysis first assesses the FW difference between the known-related and unknown-related test datasets, returning *p* values larger than the cut-off acceptance value of α=0.05, and F_Score values smaller than the F_Factor values. This finding showed the difference’s similarity between the FW values of datasets that were related to the same domain of the model, regardless of whether the datasets were presented to the model during training. However, for the difference of FW values between the known-related and unknown-unrelated test sets in Scenario 7, the *p* value was smaller than the cut-off acceptance value of α=0.05, and the F_Score value larger than the F_Factor value. Therefore, the null hypothesis is rejected, meaning there is no FW mathematical relationship when the test dataset is unknown and unrelated to the model.

A summary of the ANOVA analysis results is given in Table 3:

Based on the results of all conducted experiments and the ANOVA analysis, we could establish the following points:

1. Scenarios 1, 2, and 6 indicate that there is a mathematical relationship between the weights of a model and the features of datasets that are related to the model, regardless of whether the datasets were used or not used in training the model.
2. Scenarios 3, 4, and 5 show that there is no relationship between the weights of a model and the features of datasets that are unrelated to the model.
3. Scenarios 6 and 7 reveal that the behaviours of FW difference between related datasets are similar, but no similarity of FW difference behaviours is observed between related and unrelated datasets.

## 5. Discussion

Here, we interpret the FW relationships in the ANOVA test, as shown in Table 3, according to the three tested categories.

Firstly, we started with researching the effect of the size of the training dataset on the assumed mathematical FW relations between the model’s weights and the features of the training datasets. We linked the features of the training datasets with the model’s weights to obtain the FW values. As a result, we found that the null hypotheses failed to be rejected in both scenarios. That reflected the assumption that a relationship exists between the trained model’s weights and the features of any input dataset as long as it was presented to the model during the training process.

Secondly, we investigated the effect of the tested dataset’s types on the assumed relationship. The FW values are generated with test datasets that were never presented to the model earlier during the training process. The assessment was applied throughout the three scenarios in Scenario 3, 4, and 5, which showed the effect of unknown-related and unknown-unrelated test datasets on the FW values. As a result, we found that the null hypotheses were rejected in all three scenarios. Hence, the mathematical relationship between the trained model’s weights and the features of the test dataset does not exist as long as the test dataset is never presented to the model during the training process.

Finally, we compared the mathematical difference of FWs between the known and unknown images in the third category. The comparison was achieved by assessing the difference between two sets of FW values generated with the training dataset and an unknown dataset. The unknown dataset and the training dataset belong to the same labelled class in the sixth scenario, and not the same class in the seventh scenarios. Comparing the two FW values indicates how close the images’ features are to each other. This can be understood on the logic that two images that belong to the same classified label must share similar features [33]. We assessed the means of differences in the FW values of similar featured images in the sixth scenario. The ANOVA analysis failing to reject the null hypothesis proved that the FW difference throughout all trained model’s related classes is within the same mathematical range. This can be further interpreted as showing that those images belonging to the same classified, labelled class share similar features in FW mathematical ranges. However, the rejection of the null hypothesis in the seventh scenario supported the assumption that unknown-unrelated image’s FW values do not lie in the same mathematical ranges as that of the known-related images used in training.

Based on the experiments in the second category, we conclude that the relationship between the model’s weights and any unknown tested images’ features does not exist by rejecting all related null hypotheses. Moreover, the experiment results confirm a relationship between the model’s weights and the known-related training images’ CNN/VGG16 features as noted in the first category. Hence, the results support our assumption that a mathematical relationship exists between the model’s weights and the features of known-related images. In addition, the difference in FW values between the known-related and unknown-related test datasets to the related models show a similar close mathematical range in the sixth scenario. These findings indicate that the learning processes run similarly and generate values related only to each dataset used in training and validation. However, in the seventh scenario, a significant mismatch is recognised between the FW difference of dataset that is used in training and dataset that is both not used in training and not related to the same domain. This emphasises that the model’s weights are precisely related only to the known trained dataset, regardless of the size of the dataset. Finally, it approves the assumption that the weights are learnt from, particularly, the specifically related training dataset. We also find that any unknown dataset that belongs to the same domain of the model will generate FW values within a similar range as that of known datasets. However, the ranges are significantly different for all models when applied with datasets not belonging to the same domains of the training datasets.

A model’s learnt weights have been produced based on the features of each training dataset. Applying this to images with similar features allows the model to recognise this similarity when the model’s learnt weights are developed to recognise a particular set of features. However, in model evaluation, the model’s accuracy depends on its classification ability of new objects. Therefore, a model’s recognition ability depends on the closeness of features between the training and testing objects. Applying a dot-product operation to the test object’s features and the model’s weights can specify the uniqueness of each model. Calculating the differences between the FW values (of trained and new datasets) can be used to evaluate how much the images’ features are close to each other. The model’s weights are generated according to the images’ features of a particular training dataset. Therefore, linking the weights with the new related object’s features may determine the closeness between those features. As a result, the model can sense this closeness by applying this concept even before the actual prediction process.

One of the applications of this assumed mathematical FW relationship is to speed up the training and prediction in a complex multi-model system based on CNNs. For example, a pre-processing step using the FW relationship could decide which sub-model a new object should be applied to for training and prediction, without being tried on all models. It could also be used potentially to simplify the network training significantly if we could determine the actual mathematical relationship between the features of objects and the weights of the model. We have not investigated the actual FW relationship itself in this research, as the work we described in the paper is only an empirical proof of the assumption. It will be our next target in future research.

It is noteworthy to raise the awareness of the overfitting problem that would usually affect the prediction process when performing training on a small-sized training dataset [39]. As we have used small-sized datasets for training in the first category of experiments, it could result in the models being overfitted potentially. Therefore, it is important to avoid overfitting by tuning the hyperparameters in training carefully. However, we concentrate on proving the FW relationship assumption in this study, not generating a practical recognition model. Therefore, model overfitting is a less concern for us.

## 6. Conclusion and Future Work

This paper proved the assumption of an existing mathematical relationship between an input image’s extracted features (excitations) and a related CNN model’s learnt weights empirically. This relationship was generated based on the extracted features of all input images used in training and the trained model’s learnt weights. This approval may be considered the first pre-processing step in building an accurate recognition model in the future.

Moreover, we confirmed the resemblance in the ranges of FW mathematical differences between the images related to the same distinguished class as the images used in training the model. This supports the existence of a mathematical relationship overall between the features of images belonging to the same class as that of the training dataset and the trained model’s excitation weights. This resemblance will be potentially very useful in machine learning applications, for example, in developing feature vector comparison techniques or algorithms that support recognition based on the distance of the FW vectors. This distance analysis approach may offer clues in improving the model’s learning techniques to enforce multi-modality learning instead of performing full dataset training.

Our next step will be searching for possible learnt parameter patterns in a trained model according to the input images’ features. These patterns would significantly reduce the needed computations for performing the training from scratch whenever new classes are added to the image recognition system.

## Figures and Tables

**Table 1:** Summary of all experiments conducted in three categories (Cat.). Cat. 1 and 2 test the effects of the training dataset (TrDset) sizes and test dataset (TsDset) types on the learnt weights. Cat. 3 is for testing the mathematical difference between the training and testing datasets on the weights learnt during the model’s learning process.

Cat.	Scenario	Purpose	TrDset Size	No. of Models	TsDset Type	No. of TsDsets	TsDset Size
1	1	Effect of TrDset sizes	20	11	Known-Related	11	20
	2		10,000 for M1 8000 for M2 1300 for M3	3	Known-Related	3	400
2	3	Effect of TDset types	10,000 for M1	1	Unknown-Unrelated	3	200
	4		10,000 for M1 8000 for M2 1300 for M3	3	Unknown-Related	3	400
	5				Unknown-Unrelated	3	400
3	6	Effect of training and test datasets	10,000 for M1 8000 for M2 1300 for M3	3	Known-Related, Unknown-Related	3	200
	7				Known-Related, Unknown-Unrelated	6	200

**Table 2:** ANOVA test results of seven scenarios in three categories. It shows the effect of the training dataset sizes and the test set types on the FW relationship, and the closeness of FWs between the related and unrelated test sets to the known training dataset.

Cat.	Scenario	Trained Model	TrDset Size	TsDset Type	TsDset Size	F_Score	Critical F_Factor	Null Hypothesis (Accept or Reject)
1	1	Model_K (K = 1, … ,11)	20	Known-Related	20	1.51	1.87	Accepted
	2	Model_1 Model_2 Model_3	10 k 8 k 1.3 k	Known-Related	400	2.07	2.22	Accepted
2	3	Model_1	10 k	Unknown-Unrelated	200	77.10	3.01	Rejected
	4	Model_1 Model_2 Model_3	10 k 8 k 1.3 k	Unknown-Related	400	7.82	2.22	Rejected
	5	Model_1 Model_2 Model_3	10 k 8 k 1.3 k	Unknown-Unrelated	400	12.96	2.22	Rejected
3	6	Model_1 Model_2 Model_3	10 k 8 k 1.3 k	Known-Related vs. Unknown-Related	200	1.84	2.22	Accepted
	7	Model_1 Model_2 Model_3	10 k 8 k 1.3 k	Known-Related vs. Unknown-Unrelated	200	9.76	1.79	Rejected

**Table 3:** Description of ANOVA analysis results for seven scenarios in three categories.

Cat.	Scenario	Null Hypothesis (Reject or Accept)	ANOVA Result	Interpretation of the ANOVA Result
1	1	Accepted	No significant difference between the FW values with small training and known-related test datasets.	(a) Similar mathematical relationship exists between the features of known-related training datasets and trained model’s weights, regardless of whether the model is trained with a large or small training dataset.
	2	Accepted	No significant difference between the FW values with large training and known-related test datasets.	
2	3	Rejected	Significant difference exists between the FW values for a model with large training and unknown-unrelated test datasets.	(b) No similar mathematical relationship exists between the features of test dataset and weights of trained model as long as the test dataset is unknown to the model.
	4	Rejected	Significant difference exists between the FW values for three models with large training and unknown-related test datasets.	
	5	Rejected	Significant difference exists between the FW values for three models with large training and unknown-unrelated test datasets.	
3	6	Accepted	No significant difference in the FW difference between known-related and unknown-related test datasets.	(c) The FW difference between known and unknown test datasets is similar as long as the test dataset is related to the model. (d) No similarity in the FW difference between the related and unrelated test datasets.
	7	Rejected	Significant difference exists in the FW difference between known-related and unknown-unrelated test datasets.	

## Data Availability

Dataset and Python code are available on GitHub publicly.
